# Immunobiological effects of lipopolysaccharide derived from *Helicobacter pylori* and influence of a proton pump inhibitor lansoprazole on human polymorphonuclear leukocytes

**DOI:** 10.1007/s12223-024-01188-7

**Published:** 2024-08-17

**Authors:** Yoji Koshibu, Tsuneyuki Ubagai, Yusuke Yoshino, Yasuo Ono

**Affiliations:** 1 Department of Microbiology & Immunology, Teikyo University School of Medicine, Kaga 2-11-1, Itabashi-Ku, Tokyo, 173-8605 Japan; 2 Faculty of Health and Medical Science, Teikyo Heisei University, Tokyo, 170-8445 Japan

**Keywords:** *H. pylori*, LPS, Polymorphonuclear leukocyte, Lansoprazole, RT-PCR

## Abstract

*Helicobacter* *pylori* colonizes the human gastric mucosa of more than half of the human population and has a unique lipopolysaccharide (LPS) structure. LPS is the most dominant and suitable pathogen-associated molecular pattern that is detected via pattern recognition receptors. Although the priming effect of *H. pylori* LPS on reactive oxygen species (ROS) production of PMNs is lower than that of *Escherichia coli* O111:B4 LPS, LPS released from *H. pylori* associated with antibiotics eradication therapy may activate PMNs and increase ROS production. In addition, we describe the effects of *H. pylori* and *E. coli* O111:B4 LPSs on gene expression and the anti-inflammatory effect of lansoprazole (LPZ) in human polymorphonuclear leukocytes. LPS isolated from *H. pylori* and *E. coli* O111:B4 alters toll-like receptor 2 (TLR) and TLR4 expressions similarly. However, LPS from *E. coli* O111:B4 and *H. pylori* caused a 1.8-fold and 1.5-fold increase, respectively, in *CD14* expression. All LPS subtypes upregulated *TNFα* and *IL6* expression in a concentration-dependent manner. Although *E. coli* O111:B4 LPS upregulated *IL8R* mRNA levels, *H. pylori* LPS did not (≦ 100 ng/mL). Gene expression levels of *ITGAM* demonstrated no significant change on using both LPSs. These different effects on the gene expression in PMNs may depend on variations in LPS structural modifications related to the acquired immunomodulatory properties of *H. pylori* LPS. Proton pump inhibitors, i.e., LPZ, are used in combination with antibiotics for the eradication therapy of *H. pylori*. LPZ and its acid-activated sulphenamide form AG-2000 suppress ROS production of PMNs in a dose-dependent manner. These results suggest that LPZ combination with antibiotics for *H. pylori* eradication reduces gastric inflammation by suppressing ROS release from PMNs.

## Introduction

*H. pylori* is a spiral-shaped Gram-negative rod bacterium that infects the human host’s stomach, and more than half of the world’s population is colonized by this pathogen, thus making *H. pylori* the most common GNR infection worldwide (Peek and Blaser 2002). Several prominent gastrointestinal diseases are related to *H. pylori* colonization in the human stomach, including gastritis, peptic ulcers, stomach cancer, and mucosa-associated lymphoid tissue lymphoma (De Falco et al. 2015). The onset or silence of symptoms and their severity depend on various pathogen-associated molecular patterns, host susceptibility, and environmental factors that allow *H. pylori* to switch between pathogenicity and commensalism.

**Fig. 1:**
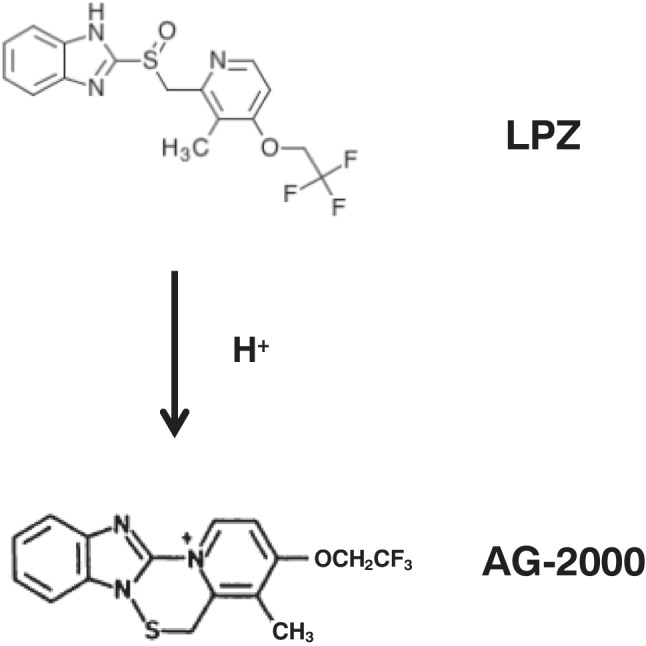
Chemical structures of lansoprazole (LPZ) and its acid-induced rearrangement product AG-2000

The main treatments for *H. pylori* infection have been a combination of broad-spectrum antibiotics, bismuth salts (Alkim et al. 2017), and proton pump inhibitors (PPIs) which render the bacteria more susceptible to antibiotics (Scott et al. 2016). Eradication of *H. pylori* has been found to be associated with improved healing rates of peptic ulcer healing and reduced risk of gastric cancer (Gisbert et al. 2012; Ford et al. 2014). The standard triple treatment of PPI, clarithromycin, and amoxicillin or metronidazole is recommended as the first-line eradication therapy for *H. pylori* infection in clinical guidelines worldwide (Malfertheiner et al. 2012; Chey and Wong 2007). Omeprazole (OPZ) and lansoprazole (LPZ) are first-generation PPIs. The interaction profiles of OPZ have been most extensively reported (Wedemeyer and Blume 2014). OPZ has a significant potential for drug-drug interactions. This is because OPZ has a high affinity for CYP2C19 and a moderate affinity for CYP3A4. On the other hand, LPZ has a lower incidence of drug interactions than OPZ. The interaction profiles of LPZ have not been studied as thoroughly as those of OPZ. In fact, no clinically significant interactions have been reported between LPZ and phenazone, diazepam, ivabradine, magaldrate, oral contraceptives, phenytoin, prednisolone, propranol, or warfarin (Wedemeyer and Blume 2014). Antiulcer benzimidazole PPI, such as lansoprazole (LPZ), and its acid-activated sulphenamide form AG-2000 (Schmarda et al. 2000) (Fig. 1) are potent and specific suppressors of urease and growth of *H. pylori*. LPZ suppresses H^+^ and K^+^ ATPase ((H^+^/K^+^)-ATPase) of stomach parietal cells and dose-dependently inhibits the urease activity in cell extracts of *H. pylori* (Nagata et al. 1993).

Some non-gastric cells, for example, polymorphonuclear leukocytes and endothelial cells, have vacuolar H^+^ATPases that incorporate acid into the extracellular space and intracellular organelles such as lysosomes. These ATPases appear to be susceptible to inhibition by PPIs. Indeed, the effect of PPIs has been reported to have antioxidant properties and effects on PMNs, monocytes, and endothelial cells that might be anti-inflammatory (Ritter et al. 1998). *H. pylori* activates gastric epithelial cells and PMNs via TLRs (Rad et al. 2009). The most prominent driver of host responses is approximately 70% of all *H. pylori* strains harbors the *cag* pathogenicity island (cagPAI) in the world (Olbermann et al. 2010). PMNs constitute the most abundant first wave of recruited immune cells to *H. pylori* infectious sites in the stomach (Petri et al. 2008). A chronic PMN-rich inflammatory response characterizes the infection, and the PMN population correlates with disease severity and digestive organ damage (Allen. 2007). Lipopolysaccharide (LPS) derived from Gram-negative bacteria activates PMN response and enhances reactive oxygen species (ROS) release, which contributes to the aggravation of inflammation. *H. pylori* LPS suppresses its evacuation from the gastric mucosa, modulating effector cells’ function and influencing the adaptive T-cell response, thus enhancing the development of chronic infections (Portal-Celhay and Perez-Perez 2006). *H. pylori* LPS is characterized by fewer and longer fatty acid residues, a lack of 4-phosphate groups, and an ethanol group linked to the glycosyl phosphate; therefore, *H. pylori* LPS shows low biological activity by host cytokine induction (Hynes et al. 2004). The endotoxicity, lethality, and pyrogenicity activities of *H. pylori* LPS are lower than those of other typical LPSs such as those from *E. coli* strains (Matsuyama et al. 2001).

However, gene expression analysis in PMNs stimulated by LPS from the *H. pylori* strain has not been reported. In this study, we employed the purified *H. pylori* LPS to elucidate their effects on PMN gene expressions and ROS production. In addition, we investigated the effect of LPZ and its acid-activated sulphenamide form AG-2000 on the ROS-producing ability of PMN.

## Materials and methods

### Reagents

The endotoxin-free Hanks’ balanced salt solution (HBSS), phosphate-buffered saline (PBS), and deionized water were purchased from Life Technology Japan (Tokyo, Japan). All reagents were checked and confirmed to be endotoxin-free, DNase-free, RNase-free, and pyrogen-free by Life Technology Japan. Furthermore, all reagents and solutions included in the commercial kits in this study were confirmed to be free from contamination using endotoxin testing, which was performed in compliance with the following guidelines: ANSI/AAMI ST72: 2011; Bacterial Endotoxins-Test methods, routine monitoring, and alternative to batch testing; USP < 85 > Bacterial Endotoxin Test (F.D.A. n.d.).

### Chemicals

All chemicals used are of pro analysis grade. Takeda Pharmaceutical (Japan) kindly provided lansoprazole (2-[[[3-methyl-4-(2,2,2 -trifluoroethoxy)-2-pyridyl]methyl]sulfinyl]-1H-benzimidazole) and AG2000 (4-methyl-3-(2,2,2-trifluoroethoxy)-5H-pyrido[1′,2′: 4, 5][1, 2, 4]thiadiazino [2, 3-a]benzimidazole-13-ium tetrafluoroborate) (Schmarda et al. 2000) (Fig. 1).

### Lipopolysaccharides (LPSs)

LPSs isolated from *Escherichia coli* O111:B4 (B4) and *Helicobacter pylori* (Hp) were purchased from Wako Pure Chemical Industries (Tokyo, Japan). LPSs were suspended in 100% dimethyl sulfoxide (DMSO) (endotoxin-free) to prepare a 100 μg/mL stock solution, which was adjusted to various concentrations by dilution with HBSS (Ubagai et al. 2021).

### Human blood samples

We obtained blood from 9 volunteer donors (6 men and 3 women; age 29 − 58 years: mean age, 39). Each donor donated blood more than once, and the mRNA expression levels of the 7 genes (one was the reference gene, *ACTB*) were averaged. Healthy individuals were confirmed, at the start of the study, through consent forms and interviews to be physically healthy, non-smokers who were not taking any medication were informed about the study’s purpose, and consent was obtained from all participants. The protocol was approved by the Ethical Review Committee at the Teikyo University School of Medicine (No. 07–104).

### PMN preparation

Human PMNs were isolated from healthy volunteers’ peripheral blood (Ubagai et al. 2008). Briefly, 20 mL of heparinized whole blood was mixed with 4.5% dextran solution and allowed to stand for 40 min at room temperature. The leukocyte-rich plasma was centrifuged at 400 × *g* on an endotoxin-free Ficoll-Paque Plus gradient (Amersham Bioscience, WI, USA) for 20 min. Hypotonic (0.2%) saline was used to lyse erythrocytes, and osmolality was restored by hypertonic (1.6%) saline. PMNs were only used in experiments if the viability was 99% or more (as assessed by trypan blue exclusion) and the purity was at least 95% (as assessed by morphological analysis). PMNs were adjusted to a final concentration of 1 × 10^7^ cells/mL in endotoxin-free HBSS.

### PMN chemiluminescence assay

Human PMNs (5 × 10^5^ cells/mL) were treated with several concentrations of LPS (final concentration, 0–1000 ng/mL), LPZ, or AG-2000 (final concentration, 0–25 µg/mL). Then, 20 µL of luminol solution (40 µg/mL) was added. The mixture was incubated at 37 °C for 60 min. The reaction mixture was stimulated with 10 µL fMLP (10^−5^ M), 20 µL zymosan A (500 µg/mL), or 5 µL PMA (500 ng/mL) for ROS production. Finally, the CL intensity was continuously measured for 10–20 min with a six-channel Biolumat LB 9505 device (Berthold, Wildbad, Germany).

### PMN stimulation with LPS

LPS, resuspended in 100% DMSO (endotoxin-free), was inoculated into PMN suspension (5 × 10^6^ cells/mL) at 10, 100, or 1000 ng/mL. Control PMNs (5 × 10^6^ cells/mL) with serum (0.2%) that were unstimulated (LPS −) were also prepared, with a final DMSO concentration of 1%. The mixtures were incubated with controls for 10 and 30 min in an environment containing 5% CO_2_. Following stimulation, PMNs were collected and washed with cold endotoxin-free PBS.

### RNA preparation

Human PMNs (5 × 10^6^ cells/mL) were washed with 1 mL of cold endotoxin-free PBS. Total RNA was extracted using the RNeasy Plus Mini Kit (QIAGEN, Germany) following the manufacturer’s instructions. The quantity and quality of total RNA samples were determined using the Agilent 2100 Bioanalyzer (Agilent Technologies, Germany).

### Complementary DNA synthesis

Total RNA was reverse-transcribed to cDNA using the SuperScript VILO cDNA synthesis kit (Invitrogen Life Technologies, CA, USA). Briefly, 1 µg of total RNA was incubated with 2.5 µM oligo (dT)_20_, 50 ng of random hexamers, and 200 U SuperScript III RT enzyme in a 20 µL reaction volume at 42 °C for 60 min, followed by 50 °C for 20 min. The reactions were terminated by heating the solution at 85 °C for 5 min.

### Quantitative real-time PCR (qPCR) analysis

The mRNA expression levels of all genes were quantified in PMNs after LPS treatment for 10 and 30 min (Ubagai et al. 2009). In gene expression analysis, the control group was untreated PMNs, i.e., intact PMNs resuspended in endotoxin-free HBSS. The *TLR* gene expression levels (GenBank accession no. MN_018643.2) in PMNs were quantified using the StepOne Real-Time PCR System (Applied Biosystems, CA, USA). Complementary DNA was amplified with SYBR Green by using the Power SYBR Green PCR Master Mix (Invitrogen, CA, USA). Briefly, qPCR was performed for *TRLs* and the housekeeping gene *ACTB* (GenBank accession no. MN_001101.1). The PCR primer sets are described in Table 1. The following cDNA amplification program was used: 95 °C for 10 min and then 40 cycles of 95 °C for 15 s and 60 °C for 1 min. All PCR reactions were performed in 20 µL reaction volumes comprising the following components: 5 µL of cDNA solution, 0.9 U Ampli *Taq* Gold DNA polymerase, 1 × reaction buffer (20 mM Tris–HCl pH 8.4, 3 mM MgCl_2_, 200 µM dVTPs (mixture of dATP, dCTP, and dGTP), 400 µM dUTP, 500 nM ROX reference dye, and 0.6 U Uralic glycosylase), and 200 nM primers. *TLR* mRNA expression levels in PMNs were normalized to the *ACTB* gene expression levels. Fold changes of PMN *TLR* mRNA levels between LPS-stimulated samples and untreated controls were determined using the Sequence Detection System (SDS) software (Applied Biosystems). Briefly, in this study, we employed the absolute quantification method for qPCR analysis. The copy numbers of each target gene were divided by the copy number of the same gene in untreated PMNs. All target gene copy numbers were adjusted relative to the number of copies of the housekeeping gene *ACTB*.

**Table 1:** Primer sets for quantitative polymerase chain reaction

qPCR primer sets		
Genes	Sequence	Amplicon size (bp)
*TLR2*	F: 5′-TCTGCTATGATGCATTTGTTT-3′	150
	R: 5′-TATTGTCAATGATCCACTTGC-3′	
*TLR4*	F: 5′-ATTTCAGCTCTGCCTTCACTA-3′	212
	R: 5′-CTTCTGCAGGACAATGAAGAT-3′	
*CD14*	F: 5′-CGCTCGAGGACCTAAAGATA-3′	243
	R: 5′-CAGACAGGTCTAGGCTGGTAA-3′	
*TNFα*	F: 5′-AGACCAAGGTCAACCTCCT-3′	194
	R: 5′-AAAGTAGACCTGCCCAGAC-3′	
*IL6*	F: 5′-AGCTATGAACTCCTTCTCCAC-3′	170
	R: 5′-GTTTGTCAATTCGTTCTGAAG-3′	
*IL8Rs*	F: 5′-GGTCATCTTTGCTGTCGTCC-3′	191
	R: 5′-CGTAGATGATGGGGTTGAG-3′	
*ITGAM*	F: 5′-AAGGTGTCCACACTCCAGAAC-3′	204
	R: 5′-GAGGAGCAGTTTGTTTCCAAG-3′	
*ACTB*	F: 5′-TTAAGGAGAAGCTGTGCTACG-3′	205
	R: 5′-TTGAAGGTAGTTTCGTGGATG-3′	

*F* forward primer, *R* reverse primer

### Statistical analysis

All *P* values were determined by the nonparametric unpaired or paired *t*-tests (two-tailed) using Excel 2011 (Microsoft Corporation, Tokyo, Japan). We considered *P* < 0.05 to be significant, and the degree of significance was indicated as **P < 0.01.

## Results

### PMN-CL assay

**Fig. 2:**
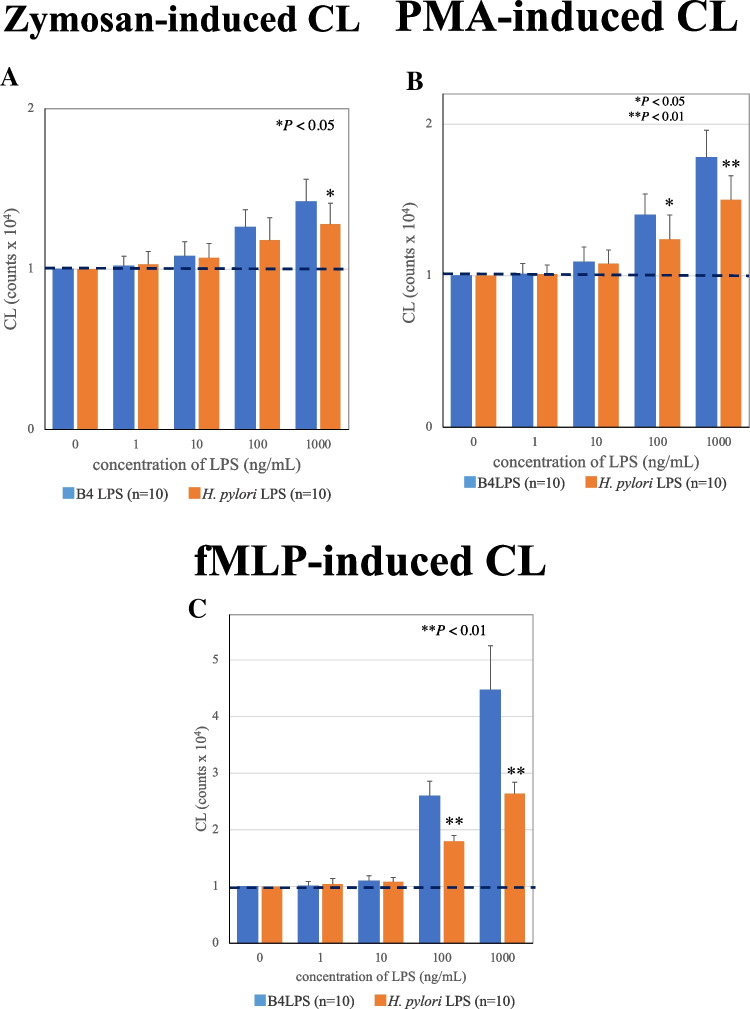
PMN-CL assay. **A** Zymosan-induced CL. **B** PMA-induced CL. **C** fMLP-induced CL. LPSs were administered at 0, 10, 100, and 1000 ng/mL. *P < 0.05 and **P < 0.01 compared to non-LPS treatment group. Error bars represent SEM. Data are representative of 10 separate experiments

The quantitative analysis of ROS generation from intact PMNs showed no difference between *E. coli* (B4) LPS and *H. pylori* LPS activation when the concentrations were under 10 ng/mL and the stimulators were zymosan (Zym), phorbol 12-myristate 13-acetate (PMA), and formyl-methionyl-leucyl-phenylalanine (fMLP). However, the two types of LPSs enhanced at concentrations above 100 ng/mL, and ROS generation of B4 LPS activation was higher than that of *H. pylori* LPS activation using three stimulators. Indeed, when fMLP was used as a stimulator, the ROS generation of *H. pylori* LPS activation was significantly lower than that of B4 LPS activation (Fig. 2A–C).

### Gene expression analysis

We did not find any significant differences in the gene expression levels of the 7 genes (Table 1) tested in PMNs between healthy men and women stimulated with LPS under the same conditions (Ubagai et al. 2015).

### Pattern recognition receptors in LPS-stimulated PMNs

**Fig. 3:**
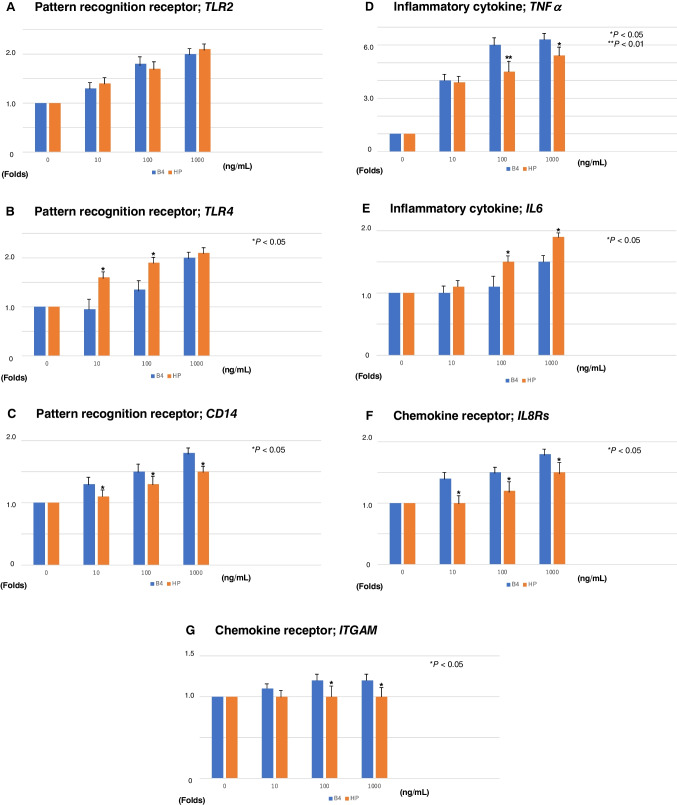
Gene expression analysis of pattern recognition receptors in LPS-stimulated PMNs. **A** mRNA expression levels of *TLR2*. **B** mRNA expression levels of *TLR4*. **C** mRNA expression levels of *CD14*. Gene expression analysis of inflammatory cytokines in LPS-stimulated PMNs. **D** mRNA expression levels of *TNFα*. **E** mRNA expression levels of *IL6*. Gene expression analysis of chemokine receptors in LPS-stimulated PMNs. **F** mRNA expression levels of *IL8Rs*. **G** mRNA expression levels of *ITGAM*. LPS was administered at 0, 10, 100, and 1000 ng/mL. All reaction tubes were incubated at 37 °C for 10 min. mRNA expression levels observed at 0 ng/mL LPS were considered as the baseline and designated as 1.0. The fold change is indicated as a ratio. *P < 0.05 and **P < 0.01 compared to healthy subjects. Error bars represent SEM. Data are representative of at least 3 separate experiments

*TLR2* gene expression levels in B4 or *H. pylori* LPS-stimulated PMNs were not significantly altered, and mRNA expression levels in PMNs were upregulated 2.0-fold at 1000 ng/mL LPS stimulation (Fig. 3A, Table 2). Similarly, B4 and *H. pylori* LPS-stimulated PMNs expressed 2.0-fold levels of *TLR4* transcript at 1000 ng/mL LPS stimulation (Fig. 3B, Table 2). A predominant difference between B4 and *H. pylori* at 10 and 100 ng/mL LPS stimulation was observed. PMNs stimulated by all LPS subtypes upregulated *CD14* expression. B4 LPS elicited a 1.8-fold increase in *CD14* expression at 1000 ng/mL; however, *H. pylori* LPS-stimulated PMNs expressed a 1.5-fold increase at same the concentration of LPS (Fig. 3C, Table 2).

**Table 2:** Average fold change of gene expression levels in LPS-stimulated PMNs related to untreated controls

Immunomodulator genes	LPS concentration (ng/mL)						
	0	10		100		1000	
Bacteria	(–)	B4	*Hp*	B4	*Hp*	B4	*Hp*
Pattern recognition receptors							
* TLR2*	1.0	1.3	1.4	1.8	1.7	2.0	2.1
* TLR4*	1.0	0.9	1.6*	1.3	1.9*	2.0	2.1
* CD14*	1.0	1.3	1.1*	1.5	1.3*	1.8	1.5*
Inflammatory cytokines							
* TNFα*	1.0	4.0	3.9	6.0	4.5**	6.3	5.4*
* IL6*	1.0	1.0	1.1	1.1	1.5*	1.5	1.9*
Chemokine receptors							
* IL8Rs*	1.0	1.4	1.0*	1.5	1.2*	1.8	1.5*
* ITGAM*	1.0	1.1	1.0	1.2	1.0	1.2	1.0

LPS was administered at 0, 10, 100, and 1000 ng/mL. All reaction tubes were incubated at 37 °C for 10 min. mRNA expression levels observed at 0 ng/mL LPS were considered as the baseline and designated as 1.0. The fold change is indicated as a ratio

^*^P < 0.05 and **P < 0.01 compared to healthy subjects

### Inflammatory cytokines in LPS-stimulated PMNs

All LPS subtypes upregulated the *TNFα* and *IL6* expression in PMNs in a dose-dependent manner. Moreover, the transcript levels of *TNFα* from B4 LPS-stimulated PMNs were higher than those in PMNs stimulated by *H. pylori* LPS. A predominant difference between B4 and *H. pylori* at 100 ng/mL LPS stimulation was observed (Fig. 3D, Table 2). In contrast, *IL6* mRNA levels of *H. pylori* stimulated by LPS were higher than that of B4 levels. A predominant difference between B4 and *H. pylori* at 100 and 1000 ng/mL LPS stimulation was also observed (Fig. 3E, Table 2).

### Chemokine receptors in LPS-stimulated PMNs

*IL8R* expression levels in B4 LPS-treated PMNs were 1.8-fold at 1000 ng/mL LPS stimulation; however, the *IL8R* mRNA level of *H. pylori* LPS-stimulated PMNs was lower than that of B4 (Fig. 3F, Table 2). The LPS-stimulated levels of *ITGAM* expression in PMNs were not significant at three points of LPS concentrations (Fig. 3G, Table 2).

### Effect of PPI and AG-2000 on PML-CL assay

**Fig. 4:**
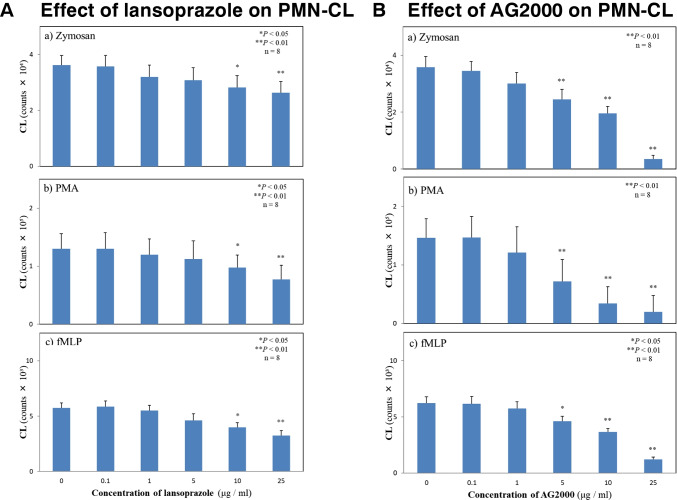
Effects of PPI on PMN-CL. **A** The effect of lansoprazole on PMN-CL. **B** The effect of AG-2000 on PMN-CL. PPIs were administered at 0, 0.1, 1.0, 5, 10, and 25 mg/mL. *P < 0.05 and **P < 0.01 compared to non-PPT treatment group. Error bars represent SEM. Data are representative of 8 separate experiments

The effect of LPZ treatment on intact PMNs and quantitative analysis of ROS generation were not any different between Zym and PMA stimulations when the concentrations were under 25 mg/mL (Fig. 4A). However, the basic component of LPZ, AG-2000, significantly suppressed the ROS generation from intact PMNs. The concentration was over 5 mg/mL (Fig. 4B).

## Discussion

*H. pylori* is one of the most common bacterial infections worldwide. The infection causes a pro-inflammatory signaling cascade, inducing the transcriptional activation and secretion of cytokines and chemokines. *H. pylori* LPS is an extremely important pathogenic and virulence factor; however, the bioactivities revealed significantly lower endotoxicity and immunological activities compared with an enterobacterial LPS (*E. coli* O111: B4-LPS). In many cases, asymptomatic *H. pylori*–infected patients likely develop very mild gastric inflammation in response to *H. pylori.* LPS released from *H. pylori* following antibiotic eradication therapy may activate PMNs. In this study, *TLR2* gene expression levels in B4 or *H. pylori* LPS-stimulated PMNs were not significantly altered and upregulated mRNA expression levels in PMNs by 2.0 folds (Fig. 3A, Table 2). Similarly, B4 and *H. pylori* LPS-stimulated PMNs expressed 2.0-fold levels of *TLR4* transcript (Fig. 3B, Table 2). In some reports, both *TLR2* and *TLR4* contribute to the early immune response against *H. pylori* infection, causing cytokine and chemokine production (Obonyo et al. 2007). *H. pylori* LPS upregulates *TLR2* expression in gastric epithelial cells through an enhanced *TLR4*-related signaling pathway via the extracellular signal-regulated kinase (ERK)-associated NF-kB pathway (Uno et al. 2007). These signaling pathways may be activated in *H. pylori* LPS-stimulated PMNs.

PMNs stimulated by all LPS subtypes upregulated *CD14* expression. B4 LPS elicited a 1.8-fold increase in expression at 1000 ng/mL; however, *H. pylori* LPS-stimulated PMNs expressed 1.5-fold at the same concentration (Fig. 3C, Table 2).

When the epithelial cell lineage was stimulated by *E. coli* or *H. pylori* LPS, *H. pylori* LPS was much less active than *E. coli* LPS and released interleukin (IL)-8 via CD14 (Kirkland et al. 1997). All LPS subtypes upregulated *TNFα* and *IL6* expression in PMNs in a dose-dependent manner. Moreover, the *TNFα* transcript levels from B4 LPS-stimulated PMNs were higher than those in PMNs stimulated by *H. pylori* LPS (Fig. 3D, Table 2). In contrast, *IL6* mRNA levels of *H. pylori* stimulated by LPS were higher than that of B4 level (Fig. 3E, Table 2). The gastric biopsies of *H. pylori*–infected patients showed elevated expression of pro-inflammatory cytokines such as TNF*α* and IFN-γ (Abdollahi et al. 2011).

After all, chronic inflammation of gastric mucosa and duodenum is induced by *H. pylori* infection because the mRNA expression levels of inflammatory cytokines *TNFα* and *IL6* are upregulated by *H. pylori* LPS similar to *E. coli* LPS (Fig. 3D, E). Although the priming effect of *H. pylori* LPS on ROS production of PMNs was lower than that of B4 LPS, *H. pylori* LPS had the ability to augment (Fig. 2).

The lipid A moieties of *H. pylori* construct characteristic structures that differ from those of *E coli*. These moieties have longer and fewer fatty acid residues and often lack the 4′-phosphate moieties found in the *E. coli* lipid A. For example, the *H. pylori* strain 206–1 mainly presented a tri-acyl type lipid A, whereas the strain NCTC 11637 mainly had the tetra-acyl type lipid A, and penta-acylated structures were also found (Fujimoto et al. 2016). In addition, CD14 on PMNs binds LPS-LBP (LPS-binding protein), resulting in TLR4-MD2 (myeloid differentiation protein 2) activation (Guerville and Boudry 2016), as a result, not a few of these conformations. In our study, the transcript levels of *TNFα* from B4 LPS-stimulated PMNs were higher than those in PMNs stimulated by *H. pylori* LPS (Fig. 3D, Table 2). In contrast, *IL6* mRNA levels of *H. pylori* stimulated by LPS were higher than that of B4 level (Fig. 3E, Table 2). According to the experimental conditions, tri-acylated lipid A (phosphate form) and Kdo-lipid A (both phosphate and ethanolamine forms) demonstrated antagonistic activity in LPS-stimulated induction of TNFα, whereas tri-acylated lipid A (ethanolamine form) was a weak agonist (Fujimoto et al. 2016). LPZ and its acid-activated derivative AG-2000 suppressed ROS production of PMNs in a dose-dependent manner (Figs. 1 and 4). These results suggest that LPZ combination with antibiotics for *H. pylori* eradication reduces gastric inflammation by suppressing ROS release from PMNs.

Future studies about the genetic and cellular mechanisms that govern *H. pylori* LPS-stimulated effects on gene expression are necessary to fully appreciate the fascinating recognition of PAMPs by pattern recognition receptors.

## Data Availability

Data sharing is not applicable—no new data is generated, or the article describes entirely theoretical research.

## Abbreviations

LPS: Lipopolysaccharide
PMN: Polymorphonuclear leukocyte
TLR: Toll-like receptor
PPI: Proton pump inhibitor
ROS: Reactive oxygen species
